# Cryogen‐Aided Chromatographic Purification of Millimole Quantities of Methane for Spectroscopic Clumped Isotope Analysis

**DOI:** 10.1002/rcm.70114

**Published:** 2026-07-01

**Authors:** Nico Kueter, Jan G. C. Meissner, Naizhong Zhang, Léna Msonnereau, Lukas Emmenegger, Joachim Mohn, Stefano M. Bernasconi

**Affiliations:** ^1^ Department of Earth and Planetary Sciences ETH Zurich Zurich Switzerland; ^2^ Institute for Petrology and Fluid Processes RWTH Aachen Aachen Germany; ^3^ Laboratory for Air Pollution/Environmental Technology Empa Dübendorf Switzerland

## Abstract

**Rationale:**

Quantum cascade laser absorption spectroscopy (QCLAS) is a fast and reliable method for analyzing the bulk (𝛿^13^C‐CH_4_, 𝛿D‐CH_4_) and clumped isotopic (∆^13^CH_3_D and ∆^12^CH_2_D_2_) composition of methane. However, precise measurements of ∆^12^CH_2_D_2_ require 0.5 to 1.8 mmol of purified methane (equivalent to 15 to 40 mL at STP). We present a cryogen‐aided preparative gas chromatography (GC) method with 1 h cycle time that quantitatively separates methane from complex gas mixtures containing N_2_, O_2_, Ar, CO_2_, H_2_O, and volatile higher alkanes.

**Methods:**

The method employs two sequential Carboxen 1000 columns, precooled to −10°C and subsequently ramped to 150°C in two heating steps, coupled with cryofocusing on charcoal and cryocollection on silica gel. The method performance was evaluated using a well‐characterized in‐house methane reference gas, either diluted in air–He matrices or stored in Exetainer vials for up to 3 weeks. Purification tests on thermogenic and biogenic methane samples assessed the repeatability of the purification method across different natural gases.

**Results:**

Cooling of the GC columns to −10°C achieves complete chromatographic purification of millimole quantities of methane from contaminant gasses. The processing introduces no measurable bulk (𝛿^13^C‐CH_4_, 𝛿‐CH_4_) and clumped‐isotope (∆^13^CH_3_D and ∆^12^CH_2_D_2_) fractionation within the 1σ repeatability of the QCLAS system. The repeatability of methane isotope measurements from natural gas samples is comparable to that obtained in tests using the well‐characterized in‐house reference gas.

**Conclusions:**

Chromatographic purification of millimole quantities of methane from synthetic and natural gas mixtures requires GC column cooling to at least −10°C to ensure sufficient separation from major air components. The developed method enables rapid and quantitative purification of up to 40 mL of methane without detectable isotope fractionation, demonstrating the applicability and robustness of the method for routine purification prior to QCLAS‐based methane clumped‐isotope analysis.

## 1 Introduction

The analysis of doubly substituted (i.e., “clumped”) methane isotopologues offers new opportunities to study the origin, fate, and formation mechanisms of natural and anthropogenic methane (CH_4_). Since the mid‐2010s, advances in high‐resolution isotope ratio mass spectrometry (HR‐IRMS) have enabled precise quantification of the relative abundances of the methane clumped isotopologues ^13^CH_3_D and ^12^CH_2_D_2_ relative to the most common isotopic variant ^12^CH_4,_ commonly reported in the ∆‐notation with respect to a stochastic distribution [1, 2]. Around the same time, it was demonstrated that ∆^13^CH_3_D and ∆^12^CH_2_D_2_ could also be measured by spectroscopic methods using quantum cascade laser absorption spectroscopy (QCLAS or TILDAS) operating in the mid‐infrared range [3, 4]. Both techniques allow the determination of clumped‐isotope ∆‐values with comparable precision and accuracy, but they differ significantly in terms of analytical run time and sample‐volume requirements [5].

A key challenge for CH_4_ clumped isotope analysis is the extremely low natural abundance of ^12^CH_2_D_2_ (~1.5 × 10^−5^ mol%), which leads to low ion‐counting rates in HR‐IRMS or weak absorption signals in QCLAS. Nevertheless, selective and precise measurement of ^12^CH_2_D_2_ is essential to fully exploit the information encoded in methane clumped isotopes [6].

A complete isotopic characterization of a methane sample by HR‐IRMS typically requires ~ 20 h, whereas QCLAS can achieve comparable precision in < 20 min [5]. Both methods require CH_4_ to be purified prior to analysis in order to avoid mass‐spectrometric or spectroscopic interferences and line broadening. Regarding sample volume requirements, HR‐IRMS can analyze both ∆^13^CH_3_D and ∆^12^CH_2_D_2_ from CH_4_ volumes as low as 2 mL,^1^ whereas at this sample size, QCLAS can achieve sufficient accuracy only for ∆^13^CH_3_D [4, 5, 7].

For QCLAS, achieving equivalent precision for ∆^12^CH_2_D_2_ requires 10–15 mL of pure CH_4_ [5]. As HR‐IRMS is currently the more widely adopted technique, the majority of established gas chromatography (GC)‐based protocols are optimized to purify CH_4_ volumes well below 6 mL (Table 1; [2, 8, 9, 10, 11]) and are largely based on the UCLA purification system designed by Young et al. [2]. Here, a gas sample volume of up to 100 mL is collected in a silica gel–filled trap at liquid nitrogen (LN_2_) temperature (i.e., −196°C) and released into a GC system, sequentially equipped with a Molecular Sieve 5 Å and a HayeSep D column to separate first N_2_, O_2_, Ar, Kr from the alkanes, and then CH_4_ from the C_2+_ hydrocarbons [2, 10, 11]. Eluting gas species are detected with a thermal conductivity detector (TCD). Haghnegahdar et al. [9] uses an extended, 7.6 m long HayeSep D column, whereas Zhang et al. [8] employed two HayeSep Q columns in series in their GC system. For their spectroscopic study on microbial CH_4_ bulk and clumped (∆^13^CH_3_D) isotopes, Wang et al. [12] collected sample gas on activated charcoal at LN_2_ temperatures and performed the preparative chromatography on a Carboxen 1000 column, prior to tapping off the eluting CH_4_ on silica gel at LN_2_ temperatures. All methods have in common that chromatographic separation was performed at GC temperatures between 25°C and 54°C [2, 9, 11].

**TABLE 1:** Overview of studies on chromatographic methane purification for clumped isotope analysis.

Study	CH_4_ target volume	Analysis	Sample collection and transfer prior GC separation				
			Adsorbent	Analyte release		He carrier flow	
Young et al. [2]	2–4 mL**	HR‐IRMS (P)	Si‐gel at LN_2_	Warming to 30°C, unspecified		20 mL/min	
Zhang et al. [8]	4–6 mL	HR‐IRMS (U)	Si‐gel at LN_2_	Hair dryer thawing for 1 min		11 mL/min	
Haghnegahdar et al. [9]	1.5–6 mL	HR‐IRMS (P)	Si‐gel at LN_2_	Unspecified		35 mL/min	
Sun et al. [10]	> 6mL	HR‐IRMS (U)	Si‐gel at LN_2_	30°C, heating strips for 10 min		50.8 mL/min	
Sivan et al. [11]	2–4 mL	HR‐IRMS (U)	Si‐gel at LN_2_	70°C, water bath for 5 min		30 mL/min	
Wang et al. [12]	Not specified	Spectroscopy (T)	Charcoal at LN_2_	Heating to 120°C, unspecified		25 mL/min	
This study	Up to 40 mL	Spectroscopy (Q)	Charcoal at LN_2_	70°C, water bath for 1 min		57 mL/min	
	**Chromatography**					**Methane tapping** *	
	**Column 1**		**Column 2**				
**Study**	**Type**	**Dimensions**	**Type**	**Dimensions**	**GC Temp.**	**CH**_**4**_ **elution**	**Trapping time**
Young et al. [2]	Molecular Sieve 5A	3 m, 1/8“ OD	HayeSep D	2 m, 1/8″, OD	25°C	17 min	30 min
Zhang et al. [8]	HayeSep Q	2 m, 2.2 mm ID	HayeSep Q	2 m, 2.2 mm ID	30°C	Not specified	Not specified
Haghnegahdar et al. [9]	Molecular Sieve 5A	7.6 m, 1/8“ OD	—	—	54°C	27 min	30 min
Sun et al. [10]	Molecular Sieve 5A	3 m, 1/4” OD	HayeSep D	1.5 m, 1/4” OD	25°C	31 min	35 min
Sivan et al. [11]	Molecular Sieve 5A	5 m, 1/4” OD	HayeSep D	2 m, 1/4” OD	50°C***	40 min	15 min
Wang et al. [12]	Carboxen 1000	1.5 m, 1/8” OD	—	—	30°C	Not specified	Not specified
This study	Carboxen 1000 40/60	1.5 m, 1/8” OD	Carboxen 1000 40/60	1.5 m, 1/8” OD	−10°C to 150°C	12 min	15 min

Abbreviations: ID, inner diameter; LN_2_, liquid nitrogen temperature (77 K); OD, outer diameter; P, panorama; Q, QCLAS; T, TILDAS; U, ultra.

* All studies capture methane on Si gel at −196°C.

** Based on reported methane amounts produced experimentally.

*** 40°C when Kr is present.

The purification of methane sample volumes sufficient for precise and accurate analysis of ∆^12^CH_2_D_2_ by QCLAS requires adaptations to the chromatographic purification protocol (ongoing studies; [5, 13]) to prevent insufficient separation of CH_4_ from other gases due to enhanced peak tailing. This issue was already described in the HR‐IRMS purification setups dealing with comparatively small sample volumes, where particularly N_2_ and O_2_ peak tailing into the CH_4_ peak requires a second, time‐consuming GC separation step (e.g., [10, 11]). Peak tailing and interference are only aggravated when dealing with larger sample gas volumes (longer tailing of N_2_ and O_2_ and shorter retention times for methane), but can be significantly minimized by adapting cryogenic GC column cooling, as it slows the elution of individual gases and increases separation efficiency (e.g., [14]). Hence, we developed a fast, cryogen‐aided gas chromatographic method to purify large volumes (up to 40 mL) of methane from complex gas mixtures containing air constituents (i.e., N_2_, O_2_, Ar, CO_2_) and volatile higher alkanes (C_2+_), typical for many thermogenic and biogenic methane‐rich gases.

## 2 Methods

### 2.1 Cryogen‐Aided Chromatographic Methane Purification System

**FIGURE 1:**
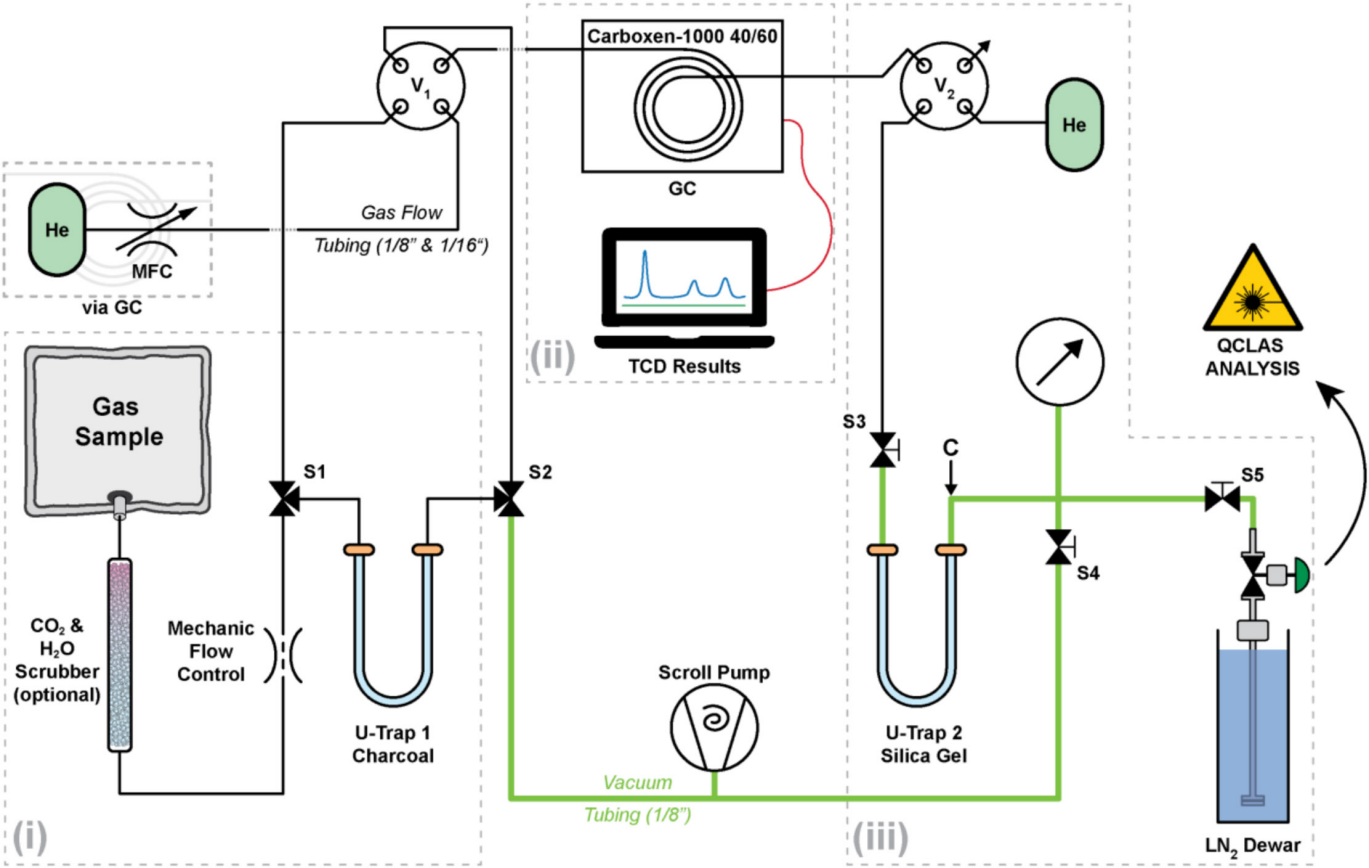
Methane purification system. (i) Sample inlet and cryofocus unit. (ii) Purification unit consisting of a gas chromatograph equipped with two Carboxen‐1000 columns for separating CH_4_ from other gas constituents. (iii) CH_4_ collection, quantification, and transfer unit.

The system shown in Figure 1 consists of three functional units:

- a sample‐inlet and cryofocus unit, used to collect and preconcentrate the gas sample
- a purification unit for separating the CH_4_ from the other gas contaminants, and
- a collection unit for collecting, quantifying, and transferring the purified CH_4_ into a storage container

Hereafter, this complete setup is referred to as the *GC purification system*.

#### 2.1.1 Sample Inlet and Cryofocus Unit

The core element of this unit is a charcoal‐filled U‐trap (U1), which is operated at −196°C (LN_2_ temperature). U1 is custom‐built from 1/4″ stainless steel tubing and filled with 2 g of activated charcoal (Merck Darco SKU: 242268). Cotton and silver wool are placed at both ends to prevent the movement of coal particles. U1 is connected via 1/8″ stainless‐steel and copper tubing to two three‐way valves (S1, S2; Swagelok SKU: B‐41XS2). Valve S2 (downstream) connects U1 either to the purification unit (Section 2.1.2) or to a vacuum line (Section 2.1.3). Valve S1 (upstream) connects U1 to either the sample inlet or a helium carrier gas supply (5.5 grade, Linde).

The sample inlet is modular and accommodates gas bags, glass or metal containers, and syringes. If required, cartridges filled with CO_2_ and H_2_O absorbents can be installed. A mechanical crimp on the hose provides precise control of the sample gas flow into U1. The relative flow rate is monitored through a pressure gauge located in the vacuum manifold (Section 2.1.3). During sample release and transfer to the purification unit, the He carrier gas is initially set to 1.4 bar and ramped up to 3.5 bar during the preparation process. When idle, S2 is closed, and U1 is kept at 1.4 bar (absolute) He pressure.

#### 2.1.2 Purification Unit

Methane separation is performed using a customized Agilent 8960 gas chromatograph (GC) equipped with a solenoid liquid‐nitrogen (LN_2_) injector nozzle (part number G3430‐60018) that is directly connected to an LN_2_ reservoir. The GC was operated at −10°C for most experiments but can be cooled to below −80°C [14].

**FIGURE 2:**
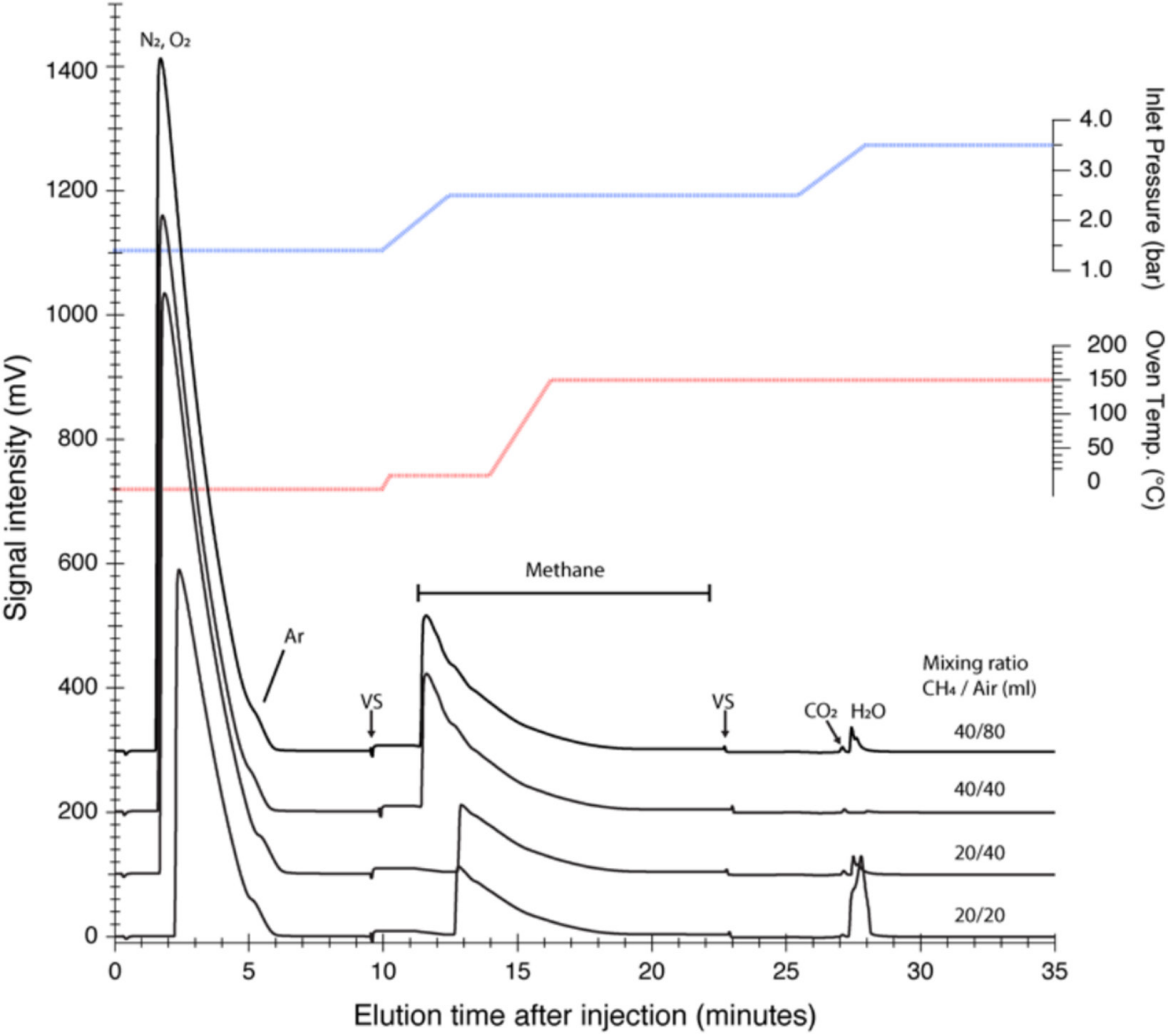
Representative chromatograms from sample purification runs using CH_4_–air mixtures diluted in 8–9 L of He. Methane is well separated from bulk air components and other impurities. Increased CH_4_ content results in earlier elution times. “VS” indicates changes in TCS response introduced by switching valve V2, at the beginning and end of the CH_4_ trapping on U2 (Figure 1; see Section 2.2). Colored lines represent the inlet pressure of the helium carrier gas (blue) and the GC oven temperature (red), which are both increased stepwise. Chromatograms are vertically offset by +100 mV increments from the zero baseline for improved visualization.

The GC system is fitted with two sequential 1.5‐m‐long, 1/8″ stainless steel Carboxen‐1000 (40/60 mesh) columns (Merck, SKU: 12380). Eluting gases are detected and quantified with a stand‐alone TCD (Model 110, SRI Systems, USA). TCD data are recorded using the SRI PeakSimple software (SRI Systems, USA). Two manually operated Vici selector valves (V1 and V2; He‐flushed valve bodies; product details) bracket the GC and TCD: V1 (upstream) either directs the He carrier gas directly into the GC (idle mode) or diverts it through U1 to sweep the adsorbate onto the first column. Valve V2 (downstream) directs the column effluent either to waste or to the collection unit (Section 2.1.3). Because the CH_4_ elution times are generally highly consistent, V2 can be safely switched approximately 90 s before CH_4_ enters the TCD without risking loss of any analyte (Figure 2; valve‐switching signal).

#### 2.1.3 Collection Unit

The collection unit consists of a second custom‐built 1/4″ U‐trap (U2), connected via 1/8″ metal tubing to the purification unit and a silica gel–filled storage container used for sample transfer to the QCLAS trap. U2 is similar in design to U1 but packed with a milder adsorbent, silica gel (Technical Grade 60—Roth, SKU: 9833.1). The reason for choosing different adsorbents, charcoal for U1 and silica gel for U2, is the fact that for U1 due to higher sample flows and volumes, a stronger retention is required but still quantitative desorption can be achieved by purging.

In contrast, passive CH_4_ desorption from charcoal traps is expected to fractionate CH_4_ isotopes, especially affecting bulk D/H and ^13^C/^12^C isotope ratios [13]. Silica gel used in trap U2 shows minimal effects on both bulk and clumped isotopic composition during passive desorption (tested adsorbents AC‐1 and SG‐2 in Kueter et al. [13]).

The vacuum manifold is isolated by a Swagelok three‐way valve (S2, connection to the sample inlet and cryofocus unit, Section 2.1.1) and three Swagelok diaphragm valves (S3‐S5, Part #SS‐DSS4), connecting it to the purification unit (valve S3; Section 2.1.2), the vacuum pump (valve S4), and the storage container for the purified sample (valve S5). The design of the storage containers is explained in Kueter et al. [13]. The vacuum manifold was operated at 10^−3^ mbar with a rotary vane pump (Duo Line, Pfeiffer Busch Group). Pressures are monitored with pressure transducer (Series M5, 0–10 bar, Keller Pressure AG). The total internal volume between S3, S4, and S5 is calibrated to 16.9 cm^3^ at laboratory temperature (23°C ± 1°C), enabling volumetric quantification of the collected CH_4_ amount.

### 2.2 Operation and Sample Processing

Samples were introduced into the GC purification system via gas bags, syringes, or sealed containers. Prior to sample processing, the GC column is cooled to −10°C. By actuating valves S1 and S2, the inlet is connected via U1 to the vacuum line and evacuated to 10^−3^ mbar. Once the baseline pressure is reached, U1 is cooled to −196°C with LN_2_ for at least 5 min. The sample valve is then opened, and the gas enters the sample‐inlet and cryofocus unit at approximately 550 mL min^−1^. During sample transfer, the line pressure is monitored with the Keller pressure transducer. For samples diluted in He, the line pressure is kept below 50 mbar to avoid CH_4_ breakthrough. For samples not diluted in He (or any other matrix gas noncondensable in LN_2_), the line pressure must remain at baseline. Transferring an 8‐ to 9‐L gas sample from a Kynar bag typically requires 15–17 min.

Once the sample is collected in U1, the connection to the vacuum line is closed (S2), and S1 and V1 are actuated to direct the He carrier gas (57 mL min^−1^) through U1 towards the purification unit. The LN_2_ Dewar is then replaced with a 70°C water bath to heat up U1 for around 30 s, releasing the CH_4_ analyte along with co‐adsorbed gas species (N_2_, O_2_, etc.). This desorption step represents the start of the GC cleaning procedure, and the timings given below are relative to it. Desorption typically causes a temporary pressure increase of 2–4 bar, as recorded by the GC internal pressure transducer. While the analyte enters the Carboxen‐1000 column, which is initially operated at −10°C, U2 is cooled to −196°C with LN_2_. The cooling phase is initiated for at least 5 min before the separated CH_4_ reaches the trap.

Two to four minutes after heating U1, the TCD detects the first gases eluting from the column. Typical chromatograms, along with the GC inlet pressure and oven temperature, are shown in Figure 2. The −10°C cooling interval is maintained for 10 min, during which N_2_, O_2_, Ar, and other highly volatile gas species (e.g., H_2_, CO) elute quantitatively. Ten minutes after heating U1, V2 is actuated to divert the gas stream through the cooled U2 into the vacuum line (S3 and S4 are opened; VS in Figure 2 indicates the valve switch). At the same time, the GC oven temperature is increased to +10°C (at 40°C/min), and the inlet pressure of the He carrier gas is set to 2.5 bar to mobilize CH_4_ and separate it from less volatile gases such as CO_2_, H_2_O, and C_2+_ hydrocarbons.

Depending on the injected sample volume, CH_4_ elutes 11–14 min after the onset of the cleaning procedure (i.e., heating U1), with the peak lasting up to 10 min for ≤ 40mL CH_4_. During this interval, CH_4_ is cryofocused on U2 while the He carrier gas is continuously pumped away. Fifteen minutes from the start of the procedure, the GC oven is heated to 150°C to support quantitative CH_4_ desorption and mobilize the remaining contaminants (i.e., CO_2_, H_2_O, C_2+_). At ~23 min, typically 2–4 min after the TCD signal from the eluting CH_4_ has returned to baseline, indicating quantitative CH_4_ desorption, V2 is switched back to direct the remaining volatiles to the exhaust.

Valve S3 is then closed, and the residual He is pumped away until the pressure in the vacuum line returns to its baseline pressure of 10^−3^ mbar. Subsequently, valve S4 is closed to seal the purified CH_4_ in the line. U2 is heated with a 50°C water bath for approximately 1 min, dried with a cloth, and allowed to equilibrate at room temperature for exactly 10 min. Then, a pressure reading is taken to calculate the amount of collected CH_4_.

During this equilibration period, a pre‐evacuated silica gel–filled storage container is cooled to −196°C with LN_2_ for at least 5 min. After the 10‐min equilibration, valve S5 is opened to transfer the purified CH_4_ sample into the storage container. The pressure gauge is used to monitor the transfer; a complete transfer is indicated by reaching baseline vacuum, which typically occurs after ~2 min. Nevertheless, trapping is continued for exactly 5 min before the storage container is closed. If required, multiple purification runs can be accumulated in the same container by keeping it cooled to −196°C.

After collection, the purified CH_4_ sample is ready for spectroscopic analysis with the QCLAS system [5].

### 2.3 QCLAS‐Based Bulk and Clumped Isotope Analysis

Methane bulk isotope (𝛿D‐CH_4_ and 𝛿^13^C‐CH_4_) and clumped isotope (∆^13^CH_3_D and ∆^12^CH_2_D_2_) analyses were performed using QCLAS (Aerodyne Research Inc., USA). A detailed description of the instrument configuration and analytical protocols is provided in Zhang et al. [5]. In brief, the spectrometer consists of an astigmatic Herriott multipass cell with an effective optical path length of 413 m and a total volume of 2.7 L, in which light emitted by two quantum cascade lasers (QCLs; Alpes Lasers, Switzerland) interacts with the analyte. The lasers are tuned to 1077 cm^−1^ to detect ^12^CH_3_D and ^12^CH_2_D_2_, and 1163 cm^−1^ to measure ^12^CH_4_, ^13^CH_4_, and ^13^CH_3_D.

Methane is introduced from a storage container via a Swagelok port and expanded to the multipass cell via an automated high‐vacuum gas inlet system with pneumatic valves controlled using the TDLWintel software (Aerodyne Research Inc.). This system ensures highly stable cell pressures (±0.05 dp/P). Prior to analysis, both the inlet line and the absorption cell are evacuated to < 10^−2^ Pa using a HiCube 80 Eco turbomolecular pump (Pfeiffer Vacuum AG, Switzerland).

Each analytical sequence begins with recording background spectra for 200 s with the multipass cell filled with 1500 Pa N_2_ to determine baseline‐absorption correction factors. Thereafter, the reference and sample gases are introduced sequentially into the cell at 1000 Pa and measured for 200 s each. A full sample measurement sequence requires approximately 17 min. Isotopic ratios are then calculated from the amount fractions of CH_4_ isotopologues quantified for the indicated rotational lines: ^12^CH_3_D (1076.8447 cm^−1^), ^12^CH_4_ (1163.4937 cm^−1^), ^13^CH_4_ (1163.6288 cm^−1^), ^13^CH_3_D (1163.4737 cm^−1^), and ^12^CH_2_D_2_ (1076.9698 cm^−1^). For calculation of the isotope ratios, see Zhang et al. [5].

All isotopic values (δ and ∆) are referenced to our in‐house high‐purity CH_4_ standard EP‐06 (grade 5.5; Linde Gas). The bulk isotopic composition of EP‐06 is 𝛿^13^C_VPDB_ = −43.75 ± 0.1‰ and 𝛿D_VSMOW_ = −188.7 ± 2.4‰, calibrated against University of Indiana methane standards [15] using gas chromatography–combustion isotope ratio mass spectrometry (GC‐C‐IRMS; [14]). The clumped‐isotopic composition of EP‐06 is 3.58% ± 0.02‰ for ∆^13^CH_3_D and 8.90 ± 0.23‰ for ∆^12^CH_2_D_2_, determined through high‐temperature equilibration experiments [5].

Reported uncertainties represent one standard deviation (1*σ*). The repeatability of the analytical procedure was determined by repeated analysis of reference gas EP‐06 as both reference and sample gas. At the time of experimentation, the repeatability was 𝛿^13^C‐CH_4_ ± 0.1‰, 𝛿D‐CH_4_ ± 0.04‰, ∆^13^CH_3_D ± 0.1 and ∆^12^CH_2_D_2_ ± 1.2‰ for measurements performed at 7.5 Torr cell pressure and 𝛿^13^C‐CH_4_ ± 0.1‰, 𝛿D‐CH_4_ ± 0.05‰, ∆^13^CH_3_D ± 0.1 and ∆^12^CH_2_D_2_ ± 2.3‰ for measurements performed at 3 and 4 Torr cell pressure. These repeatability values serve as performance targets for the purification protocol. All experiments were conducted within a single laboratory using the same instrumentation; hence, we report repeatability rather than interlaboratory reproducibility, in accordance with the definitions of the International Vocabulary of Metrology (VIM). All data are available in the supplement.

### 2.4 Experiments

#### 2.4.1 Purification Experiments Using EP‐06 Methane Reference Gas

Our purification procedure was tested for efficiency and potential isotopic fractionation using our in‐house reference gas, EP‐06 (for isotopic composition, see Section 2.3). Statistically significant deviations from zero indicate isotopic fractionation introduced by the purification process.

We conducted two experimental series with EP‐06. In the first series, we evaluated sample transfer from 10‐L Kynar bags (Sense Trading/MediSense, SKU: 400176). The bags were charged with 20 or 40 mL of EP‐06, mixed with 20–400 mL of natural laboratory air, and then brought to volume by dilution with 8–9 L of analytical‐grade He (purity 99.9999%; Linde). Methane and air were drawn using syringes (Codan Corp., SKU: SYR50LLS; Hamilton Corp. SKU: 86336SL) from EP‐06 gas cylinders or laboratory air, respectively. Methane impurities in a ventilated laboratory, around 2 ppm (2×10^−6^ mol mol^−1^), are far from affecting isotopic composition in EP‐06 reference gases (> 100′000 ppm CH_4_ in air). The tested CH_4_–air mixing ratios of 1, 0.5, 0.2, and 0.1 are representative of typical samples processed in our laboratory. One additional experiment was performed with 40 mL CH_4_ spiked with 20 mL Kr in 8–9 L He to evaluate the cryogenic separation efficiency of Kr from CH_4_, relevant for CH_4_ extracted from air.

Before use, the Kynar bags were conditioned by purging twice with N_2_ followed by a single purge with analytical‐grade He, with intermittent evacuation using a membrane pump to remove residual CH_4_. The bags were connected to the sample inlet of the GC purification system via a 3/8″ hose (without the addition of an H_2_O or CO_2_ scrubber) and processed following the protocol described in Section 2.2. Results are reported in Table 2.

**TABLE 2:** Bulk and clumped isotopic deviations of treated EP‐06 CH_4_ reference gas relative to the untreated reference gas.

Kynar sample bags									
CH_4_/Air (ml)	Mixing ratio	Purity	δ^13^C‐CH_4_	δD‐CH_4_	∆^13^CH_3_D	∆^12^CH_2_D_2_	*n*	QCLAS cell P (Torr)	Note
20/20	1	99.5 ± 0.3	−0.08 ± 0.08	0.57 ± 0.08	0.05 ± 0.07	1.52 ± 1.58	4	4	
20/40	0.5	99.5 ± 0.1	−0.14 ± 0.06	0.63 ± 0.15	0.04 ± 0.10	0.33 ± 3.04	4	4	
40/40	1	99.9 ± 0.1	−0.14 ± 0.04	0.53 ± 0.11	−0.08 ± 0.08	0.85 ± 1.08	4	7.5	
40/80	0.5	99.9 ± 0.1	−0.10 ± 0.07	0.59 ± 0.06	−0.06 ± 0.04	0.39 ± 1.03	5	7.5	
40/200*	0.2	99.8	−0.10 ± 0.01	1.16 ± 0.01	0.06 ± 0.01	0.26 ± 1.3	1	5	recycled**
40/400*	0.1	99.4	0.00 ± 0.01	0.71 ± 0.01	−0.01 ± 0.02	−1.06 ± 1.3	1	7.5	
**Exetainer vial**									
**Storage time**			**δ** ^**13**^ **C‐CH**_**4**_	**δD‐CH**_**4**_	**∆** ^**13**^ **CH**_**3**_ **D**	**∆** ^**12**^ **CH**_**2**_ **D**_**2**_	***n***	**QCLAS cell P (Torr)**	**Notes**
Fresh			−0.05 ± 0.05	0.55 ± 0.10	−0.06 ± 0.05	1.49 ± 1.91	2	4	One sample removed due to container leakage
One week			−0.04 ± 0.03	0.62 ± 0.05	−0.12 ± 0.04	4.31 ± 3.66	3	3	∆^12^CH_2_D_2_ not reliable due to low cell pressure
Two weeks			−0.07 ± 0.08	0.62 ± 0.02	−0.14 ± 0.06	−0.69 ± 1.90	3	4	
Three weeks			−0.01 ± 0.00	0.67 ± 0.04	−0.14 ± 0.02	0.82 ± 2.46	2	4	One sample removed due to container leakage

*Note:* Results are shown for experiments involving controlled air contamination and for CH_4_ stored in Exetainer vials for the indicated storage times. Uncertainties are standard deviations of multiple measurements and are reported at the 1𝜎 level.

* As only one sample was measured, typical QCLAS uncertainties from Zhang et al. [5] are reported.

** The sample was cryogenically recovered and remeasured with QCLAS.

The second experimental series evaluated the transfer of 20 mL of CH_4_ stored in chlorobutyl‐septum‐sealed 12‐mL Exetainer vials (Labco Ltd., SKU: 938 W, VW101) to assess methane isotopic integrity upon storage and transfer performance. The vials were evacuated to 10^−3^ mbar for 2 min before being filled with 20 mL EP‐06 (≈1600 Pa). Sets of three vials were processed using the GC purification system and analyzed via QCLAS immediately and after 7, 14, and 21 days of storage.

Methane was introduced to the sample inlet of the GC purification system by piercing the Exetainer septum with a syringe needle connected to the inlet hose via a vacuum‐tight Luer stopcock. No pressure increase was observed in the vacuum line during transfer, indicating quantitative trapping of CH_4_ in U1. Subsequent processing followed the protocol described in Section 2.2. Results are summarized in Table 2.

#### 2.4.2 Purification Experiments Using Natural Methane Samples

To test the applicability of our purification method to natural samples, we purified CH_4_ from both thermogenic and biogenic origin sources (Table 3). Samples were introduced into the purification unit by syringe injection, from Exetainer vials, or from Kynar sampling bags, and processed according to the protocol outlined in Section 2.2. These experiments were designed to verify chromatographic separation and assess the repeatability achieved when purifying natural methane from more complex gas matrices. Because these samples are part of ongoing studies, no details on experimental conditions (e.g., sampling strategy, pre‐preparation) and study purpose are provided.

**TABLE 3:** Purification of natural methane‐rich gas samples.

Natural samples	Label	δ^13^C‐CH_4_	δD‐CH_4_	∆^13^CH_3_D	∆^12^CH_2_D_2_	*n*	Note
Rock‐derived (thermogenic)	RS‐1	−34.97 ± 0.36	−117.74 ± 1.20	2.88 ± 0.09	5.99 ± 0.01	2	
	RS‐2	−35.98 ± 0.06	−139.89 ± 0.15	3.02 ± 0.03	7.37 ± 0.50	5	
Gas seep (thermogenic*)	GS‐1	−45.15 ± 0.01	−174.43 ± 0.05	4.89 ± 0.13	17.79 ± 0.25	2	446 ppm C2H6
	GS‐2	−46.30 ± 0.07	−174.95 ± 0.14	5.10 ± 0.02	18.22 ± 0.11	2	1100 ppm C2H6
Biogenic (microbial)	BIO‐1	−57.38 ± 0.17	−330.5 ± 0.14	−1.032 ± 0.12	−40.16 ± 2.41	3	
	BIO‐2	−57.12 ± 0.055	−329.9 ± 0.31	−1.153 ± 0.06	−40.53 ± 0.79	3	

*Note:* All uncertainties are standard deviations of multiple measurements and are reported at the 1𝜎 level.

* ∆‐values modified by microbial activity.

## 3 Results and Discussion

### 3.1 Chromatographic Separation

The chromatographic separation of gas species and the purification of CH_4_ are illustrated based on the experiments performed on CH_4_–air mixtures diluted in a He matrix. Representative chromatograms are shown in Figure 2, while chromatograms for other experiments are provided in the supplement.

The cryogen‐aided chromatographic separation of CH_4_ from co‐occurring gases shows overall robust performance. Elution proceeds in the order of decreasing volatility: N_2_, O_2_, and Ar elute first, within the first 2–6 min after injection into the GC (i.e., following heating of U1). The TCD signal returns to baseline after ~7 min, after which CH_4_ elutes, followed by CO_2_, H_2_O, and higher alkanes. Switching valve V2 changes the flow rate through the GC, producing a slight increase in the TCD baseline of ~10 mV (Figure 2, annotation “VS”).

After the GC is heated to +10°C and the inlet pressure is increased to 2.5 bar, 10 min after injection, CH_4_ takes another 1–2 min to start eluting for 40 mL injections; for smaller CH_4_ amounts, the retention time is longer. The CH_4_ peak exhibits pronounced tailing, with full elution requiring ~8 min. This undesirable, yet unavoidable tailing can be attributed to three main factors:

- column overload,
- moderate column‐heating conditions, and
- multiple internal‐diameter transitions within the flow path.

Regarding (i), given the large sample volumes, the Carboxen 1000 columns are inevitably overloaded with CH_4_, pushing adsorption into the nonlinear regime as pore space becomes progressively saturated. This results in asymmetric peak shapes with extended tailing due to heterogeneous, saturation‐limited desorption. Regarding (ii), tailing is amplified by the adoption of a relatively mild desorption temperature. During the initial cryogenic step (−10°C), CH_4_ is strongly retained within microporous domains. Subsequent heating to only +10°C provides just enough energy for desorption, but stronger heating would compromise the separation of CH_4_ from CO_2_, H_2_O, and higher alkanes. Finally, regarding (iii), the extra‐column dead volume contributes additional tailing. The step change from the 1/8″ OD. Carboxen column to the downstream 1/16″ OD, 0.04″ ID capillary of the TCD detector introduces expansion–contraction zones and small stagnant volumes in the fittings, which slow CH_4_ transport. These transitions were minimized where possible, but could not be fully avoided due to different internal diameters of components. Although these factors affect peak shape, they have no significant effect on the CH_4_ yield and the isotopic integrity of the analyzed CH_4_, as demonstrated in subsequent sections.

Finally, when the column is heated to 150°C and the inlet pressure is increased to 3.5 bar, residual CH_4_ is recovered from the GC column before remaining impurities (H_2_O, CO_2_, higher alkanes) elute between 25 and 30 min from the injection.

### 3.2 Methane Separation From Air Diluted in a He Matrix, Sampled From Kynar Bags

The separation of up to 40 mL of CH_4_ from up to 400 mL of natural air diluted in a He matrix is described below. Representative chromatograms and data are shown in Figure 2 and Table 2.

The nominal 20 and 40 mL volumes of CH_4_ introduced into the Kynar bags via syringes were quantitatively recovered after GC separation. Specifically, recovery of nominally 20 mL of CH_4_ yielded 20.5 ± 0.2 mL (*n* = 8), and recovery of 40 mL of CH_4_ yielded 40.5 ± 0.5 mL (*n* = 10). The slight systematic overestimate of CH_4_ volume by 0.5 mL likely stems from the combined syringe Luer and stopcock volume, which measures 0.4 mL [13] but is not accounted for in the syringe scale reading. The performance of the chromatographic separation is discussed in detail in Section 3.1 above.

QCLAS measurements of the 20‐ and 40‐mL series were performed at 4 and 7.5 Torr (5 and 10 mbar) cell pressure, both of which provide comparable analytical performance [5]. The repeatability of these measurements is better than the analytical repeatability of the QCLAS system (i.e., repeated direct injections of EP‐06), indicating that the purification procedure does not introduce measurable isotopic fractionation. All performance targets defined in Section 2.3 were met.

However, δ^13^C‐CH_4_ values for purified CH_4_ samples were 0.10% ± 0.07‰ (*n* = 19) lower than that of the standard. This deviation is within the QCLAS analytical repeatability (±0.1‰). Notably, this small offset is absent in the Exetainer experiments (Section 3.2), suggesting that it may be related to CH_4_ handling in Kynar bags. Regardless, the high reproducibility and small magnitude of this offset would allow a simple linear correction if required.

In addition, δD‐CH_4_ measured after purification showed a systematic increase of +0.62, ±0.16‰ (*n* = 19; or +0.59 ± 0.1‰ if the recycled 40 mL CH_4_/200 mL air experiment, showing apparent D/H fractionation, is removed; see Supplementary Information). This remarkably reproducible offset might be related to cryogenic collection of CH_4_ on silica gel after cleaning. The observed offset is consistent with our previous study on adsorbent materials, which demonstrated that δD‐CH_4_ is highly susceptible to fractionation during cryogenic adsorption–desorption [13]. A characteristic enrichment of ~0.6‰ is typical for silica gels [13], and this small, systematic fractionation can be corrected if needed. In contrast, activated charcoal and zeolite molecular sieves cause much larger isotope fractionations of up to 5‰ after passive desorption at room temperature ([13], their Table 2).

The ∆^13^CH_3_D value of EP‐06 was reproduced with high fidelity, showing no significant deviation (∆^13^CH_3_D = −0.01 ± 0.09‰; *n* = 19). The ∆^12^CH_2_D_2_ values, in turn, showed a mean deviation of +0.63 ± 1.67‰ (*n* = 19). However, spectroscopic analysis of ^12^CH_2_D_2_ is highly sensitive to QCLAS cell pressure, and analytical repeatability worsens with smaller CH_4_ volumes. We obtained ∆^12^CH_2_D_2_ = +0.9 ± 2.3‰ at 4 Torr and ∆^12^CH_2_D_2_ = +0.6 ± 1.0‰ at 7.5 Torr, which is well within performance targets, with no statistically significant fractionation observed.

### 3.3 Methane Purification After Extended Exetainer Storage

The results of the isotopic measurements from the Exetainer experiments are presented in Table 2. This experimental series consisted of four sets of three Exetainer vials, each filled with 20 mL of CH_4_. Of the 12 vials, two experiments (see Supplementary Information) were discarded because the CH_4_ transfer from U2 failed due to leakage of the receiving container. In addition, the 1‐week storage series was inadvertently measured at a cell pressure of 3 Torr, which resulted in significantly increased uncertainties for ∆^12^CH_2_D_2_ and a systematic (but not significant) shift toward more positive values. For this reason, the ∆^12^CH_2_D_2_ values from the 1‐week series were excluded from further evaluation. In summary, the storage time of CH_4_ in the Exetainer vials had no significant effect on its isotopic composition, as all isotope values were indistinguishable within uncertainty from those of freshly loaded CH_4_. The carbon isotope composition of EP‐06 was consistently reproduced across all experiments, yielding an average δ^13^C‐CH_4_ value of −0.04% ± 0.02‰. As discussed in Section 3.2, δD‐CH_4_ values showed a systematic increase of 0.62 ± 0.05‰, which is attributed to an adsorbent‐related isotope effect. The ∆^13^CH_3_D values revealed a small yet systematic fractionation of −0.11 ± 0.04‰, which remains within the performance target of ±0.15‰. The cause of this slight deviation is unclear at present. The ∆^12^CH_2_D_2_ values for the fresh, 2‐week, and 3‐week storage experiments are, on average, identical to those of untreated EP‐06 (∆^12^CH_2_D_2_ = 0.53 ± 1.12‰). This finding is consistent with previous experiments on the 𝛿^13^C‐CH_4_ fidelity of CH_4_ stored in Exetainers [16].

### 3.4 Kr Separation

The low atmospheric CH_4_ concentration (around 2 ppm) requires processing several hundred or even thousand liters of air to collect a sufficient amount of CH_4_ for clumped isotope analysis [9, 11]. As a side product, atmospheric Kr (1 ppm) is concentrated together with methane at a 2:1 ratio due to similar condensation temperatures of Kr (−153.4°C) and CH_4_ (−161.5°C). The removal of Kr is critical for subsequent isotopic measurements, regardless of the analytical method. Espic et al. [17], working on radiocarbon in CH_4_, suggested that full Kr separation may be achieved with longer column length and GC cooling. We tested this by spiking a Kynar bag containing 40 mL CH_4_ in an 8‐ to 9‐L He matrix with 20 mL Kr and lowering the initial GC temperature to −40°C; however, CH_4_ and Kr could not be separated under these conditions (Supplementary Information). This suggests that the Carboxen 1000 column is not suitable for purifying atmospheric CH_4_ concentrates. Haghnegahdar et al. [9] report sufficient Kr–CH_4_ separation using a 1/8″ diameter 7.6‐m‐long 5 Å molecular sieve column heated to 54°C. Their samples were previously processed cryogenically on a manifold to remove O_2_, N_2_, and some Kr prior to final chromatographic separation. Similarly, Sivan et al. [11] report Kr separation from a 70‐mL preconcentrated atmospheric air sample containing 2 mL of CH_4_ at 40°C, using consecutively connected Molecular Sieve 5A and HayeSep D columns. As methane clumped isotope measurements from ambient air are currently not a focus of our work, no additional attempts were taken to separate Kr.

### 3.5 Natural Samples

#### 3.5.1 Source‐Rock‐Derived Methane

**FIGURE 3:**
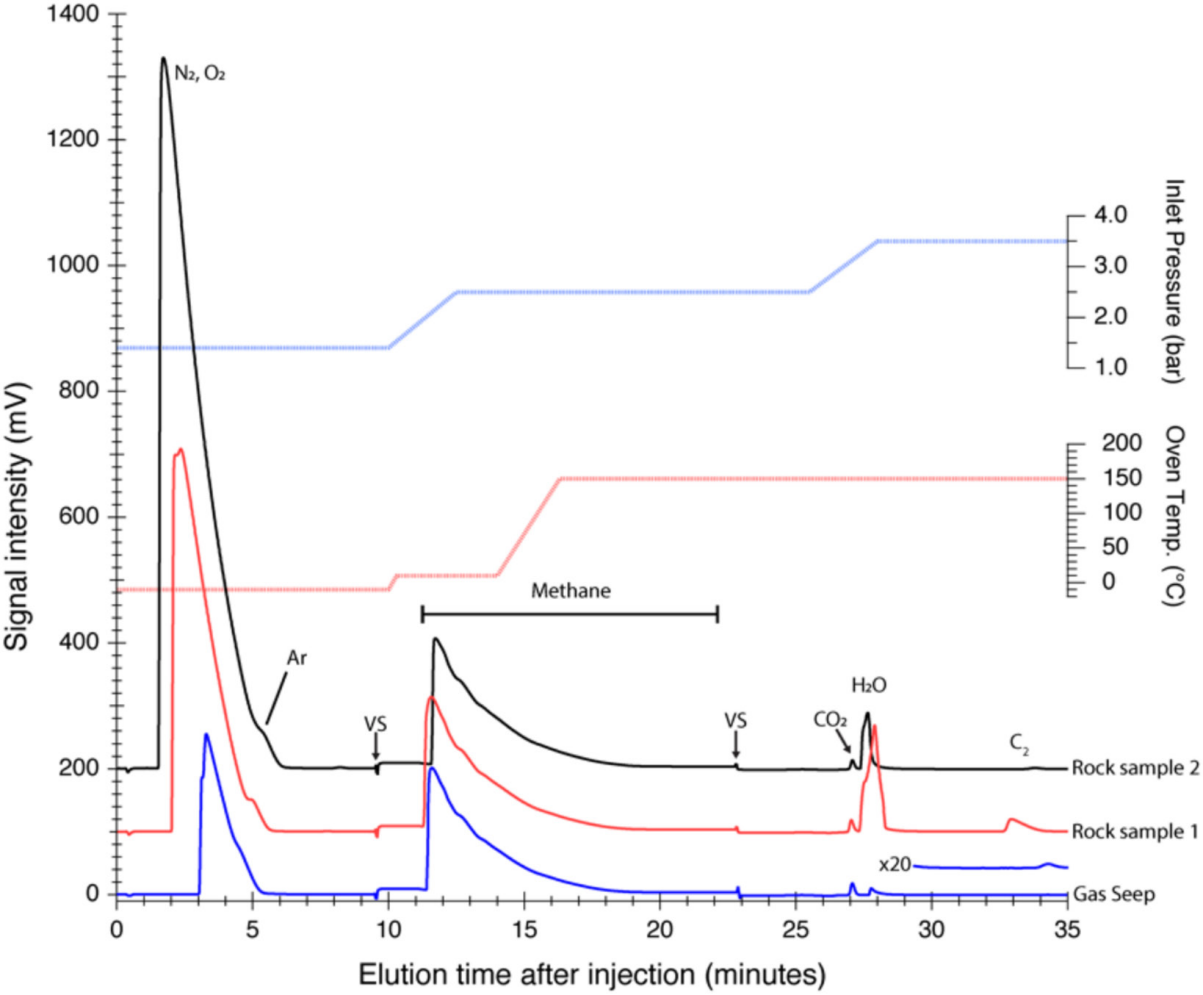
Representative chromatograms for the purification of CH_4_ recovered from source rocks and a natural gas seep. Source rocks (RS‐1, black; RS‐2, red). The recovery process inevitably introduces minor air contamination that must be removed. The samples also contain varying amounts of volatile higher alkanes. The chromatogram shows C_2_ hydrocarbons eluting at ~33 min. Natural gas seep (blue): Sampling resulted in minor air contamination. This gas sample also contained 1100 ppm ethane, visible as a small peak eluting at ~34 min (see 20× magnification). “VS” marks transient changes in TCD response caused by switching valve V2. Dashed lines indicate the ramping of the GC oven temperature and He carrier‐gas inlet pressure. The chromatogram of RS‐1 and RS‐2 is offset vertically by +100 mV for clarity.

We demonstrate the repeatability of our purification method on CH_4_‐rich gas extracted through acid digestion from organic carbon–rich source rocks (limestones and dolomites). Two rock samples (RS) from different sites were tested: CH_4_ was recovered from two aliquots of RS‐1 and five aliquots of RS‐2, yielding a total of seven purification runs and corresponding QCLAS analyses. CH_4_ recovery from source rock inevitably introduces small amounts of air, which contaminate the extracted natural gas. Besides CH_4_, the recovered gas mixtures contain large amounts of CO_2_ (predominantly from carbonate dissolution), volatile higher alkanes (C_2+_), and H_2_S. Prior to chromatographic separation, excess CO_2_ and H_2_S are quantitatively removed through an additional amine‐based gas treatment (sweetening) and recovery step (Kueter, Meissner, et al., in prep.). Following this, the sweetened gas mixture was recovered in Kynar bags in an 8‐ to 9‐L He matrix and then purified following the protocol outlined in Section. 2.2. Representative chromatograms are shown in Figure 3. They closely resemble the dilution experiments using the EP‐06 reference gas (Section 3.2), except for an additional peak eluting at 33 min, attributed to a C_2_ hydrocarbon, likely ethane. No higher‐chain hydrocarbons were detected.

Over the course of 3 months of sample preparation, purification, and measurement, the repeatability of isotopic values for rock samples from the same collection site was excellent:

For RS‐1 (*n* = 2) we obtained: 𝛿^13^C‐CH_4_ = −35.0 ± 0.4‰, 𝛿D‐CH_4_ = −117.7 ± 1.2‰, ∆^13^CH_3_D = 2.9 ± 0.1‰, and ∆^12^CH_2_D_2_ = 5.99 ± 0.01‰.

For RS‐2 (*n* = 5) we obtained: 𝛿^13^C‐CH_4_ = −36.0 ± 0.1‰, 𝛿D‐CH_4_ = −139.9 ± 0.2‰, ∆^13^CH_3_D = 3.02 ± 0.03‰, and ∆^12^CH_2_D_2_ = 7.4 ± 0.5‰.

The consistently small standard deviations demonstrate that neither the amine‐based gas treatment nor the chromatographic purification introduced significant uncertainty or compromised repeatability. Preserving the isotopic identity of CH_4_ extracted from source rock upon acid digestion and CO_2_ removal was tested but will be published elsewhere (Kueter, Meissner, et al., in prep.). These results show that our GC purification system and protocol are well suited for cleanly separating CH_4_ from complex, source rock–derived natural gas mixtures.

#### 3.5.2 Methane From Natural Gas Seeps

Methane‐rich gas samples were collected from natural gas seeps and stored in glass containers sealed with two threaded Teflon valves. Aliquots of 50 mL were collected from these containers using a gas‐tight syringe. The gas samples were then directly injected into the GC purification system and processed according to the protocol outlined in Section 2.2. Each gas sample (GS) was processed and analyzed in duplicate. Independent GC‐FID analysis revealed the presence of ethane at 446 ppm for GS‐1 and 1100 ppm for GS‐2.

The results are presented in Table 3, and a representative chromatogram is shown in Figure 3. The chromatograms are comparable to those obtained from the EP‐06 dilution experiments and the source rock–derived natural gas. For GS‐2, a weak ethane signal appears at ~34 min (Figure 3), indicating a detection limit for C_2_H_6_ of approximately 1000 ppm C_2_H_6_ in our setup. Accordingly, after CH_4_ elution, the system should be baked at 150°C for 30 min to fully remove traces of C_2+_ hydrocarbons. The isotopic compositions of the gases are highly repeatable:

For GS‐1 (*n* = 2) we obtained: 𝛿^13^C‐CH_4_ = −45.15 ± 0.01‰, 𝛿D‐CH_4_ = −174.43 ± 0.05‰, ∆^13^CH_3_D = 4.89 ± 0.13‰, and ∆^12^CH_2_D_2_ = 17.79 ± 0.25‰.

For GS‐2 (*n* = 2) we obtained: 𝛿^13^C‐CH_4_ = −46.30 ± 0.7‰, 𝛿D‐CH_4_ = −174.95 ± 0.14‰, ∆^13^CH_3_D = 5.10 ± 0.02‰, and ∆^12^CH_2_D_2_ = 18.22 ± 0.11‰.

The consistently small standard deviations indicate that neither the sampling procedure nor the chromatographic purification introduced measurable uncertainty or compromised repeatability. These results confirm that the GC purification system is well suited for separating and purifying CH_4_ from natural gas seeps.

#### 3.5.3 Biogenic Methane

Two aliquots of biogenic CH_4_ (BIO‐1 and BIO‐2) were subsampled from a gas cylinder containing biogas from the Volketswil (Switzerland) organic waste gasification plant. The plant offers several purification steps (dehumidification, desulfurization, active charcoal), including an amine‐based gas treatment to remove most CO_2_, achieving a CH_4_ purity > 96%. Before use, the biogenic CH_4_ was further purified by flushing the gas through an Ascarite/Mg (ClO_4_)_2_ trap. The subsamples were collected in 11.5 cm^3^ Swagelok stainless steel containers at 4 bar pressure and purified separately with the GC purification system. Each purified sample was then sequentially measured three times by cryogenic collection and re‐expanding into the QCLAS system. Also, for these biogenic gas samples, the isotopic measurements are highly repeatable (Table 3):

For BIO‐1 (*n* = 2) we obtained: 𝛿^13^C‐CH_4_ = −57.38 ± 0.17‰, 𝛿D‐CH_4_ = −330.49 ± 0.14‰, ∆^13^CH_3_D = −1.03 ± 0.12‰, and ∆^12^CH_2_D_2_ = −40.16 ± 2.41‰.

For BIO‐2 (*n* = 2) we obtained: 𝛿^13^C‐CH_4_ = −57.12 ± 0.05‰, 𝛿D‐CH_4_ = −329.95 ± 0.31‰, ∆^13^CH_3_D = −1.15 ± 0.06‰, and ∆^12^CH_2_D_2_ = −40.53 ± 0.79‰.

The overall small standard deviations indicate that neither the subsampling procedure nor the chromatographic purification adds significant uncertainty or degrades repeatability. These results confirm that the established GC purification system is well suited for separating and purifying biogenic CH_4_ samples.

## 4 Conclusion

High‐precision spectroscopic measurements of ∆^12^CH_2_D_2_ by QCLAS require comparatively large volumes of purified CH_4_ (typically 15–40 mL STP). Processing such volumes from air‐contaminated samples can exacerbate peak tailing and complicate baseline separation to remove interferants; however, these effects can be effectively mitigated by operating the GC columns at sufficiently low temperatures, as implemented here. Using two consecutively connected Carboxen 1000 columns operated between − 10°C and +150°C, we achieve rapid, quantitative separation of CH_4_ volumes up to 40 mL STP from complex synthetic and natural gas mixtures. Full purification requires only about 1 h cycle time and is applicable to air‐contaminated samples with CH_4_ concentrations > 10%.

The developed purification method is capable of preserving the bulk and clumped isotopic compositions across a wide range of sample types, including CH_4_ diluted in He–air matrices, Exetainer‐stored CH_4_, and natural CH_4_ from thermogenic and biogenic sources, and reproducing results within the analytical repeatability of the QCLAS system. No statistically significant fractionation was observed for ∆^13^CH_3_D or ∆^12^CH_2_D_2_, demonstrating that the GC purification system performs well without compromising isotopic integrity. Methane recoveries were consistently quantitative, and isotopic repeatability remained stable across variable injection volumes, storage times, and sample matrices. Even though the system failed to separate Kr from CH_4_, even with cryogenic cooling to −40°C, this limitation does not affect the majority of environmental or laboratory samples, as it is specific to atmospheric applications. For natural and synthetic CH_4_ mixtures containing N_2_, O_2_, Ar, CO_2_, H_2_O, and volatile higher alkanes, the presented approach offers a straightforward, reliable, and fast purification solution.

## Author Contributions

**Nico Kueter:** conceptualization, investigation, writing – original draft, methodology, validation, visualization, data curation, supervision. **Jan G. C. Meissner:** investigation, writing – review and editing, data curation, validation, methodology. **Naizhong Zhang:** writing – review and editing, investigation, validation. **Léna Monnereau:** visualization, investigation. **Lukas Emmenegger:** funding acquisition, writing – review and editing, project administration. **Joachim Mohn:** funding acquisition, writing – review and editing, project administration, resources, supervision. **Stefano M. Bernasconi:** funding acquisition, writing – review and editing, project administration, resources, supervision.

## Funding

This work was supported by the Schweizerischer Nationalfonds zur Förderung der Wissenschaftlichen Forschung (10.13039/501100001711, 200021‐200977, 206021‐183294) and the European Partnership on Metrology (10.13039/100019599, 21GRD04 isoMET).

## Supporting information




**Data S1:** Supporting Information.

## Data Availability

The data that supports the findings of this study are available in the Supporting Information of this article.

## Endnotes

^1^
All reported volumes refer to standard temperature and pressure; where 1 mmol is 22.4 mL.
